# Incidence of Raynaud's phenomenon in relation to hand-arm vibration exposure among male workers at an engineering plant a cohort study

**DOI:** 10.1186/1745-6673-3-13

**Published:** 2008-06-16

**Authors:** Mats Hagberg, Lage Burström, Ronnie Lundström, Tohr Nilsson

**Affiliations:** 1 Department of Occupational and Environmental Medicine, University of Gothenburg, Sweden; 2 Department of Occupational and Environmental Medicine, Umeå University, Umeå, Sweden

## Abstract

**Background:**

The objective of this study was to assess the incidence of Raynaud's phenomenon in relation to hand-arm vibration exposure in a cohort consisting of male office and manual workers.

**Methods:**

The baseline population consisted of 94 office and 147 manual workers at an engineering plant. Raynaud's phenomenon (RP) was assessed at baseline and at follow up (at 5, 10 and 15 years). A retrospective and a prospective cohort analysis of data were done. Hand-arm vibration exposure dose was defined as the product of exposure duration and the weighted hand-arm vibration exposure value according to ISO 5349-1.

**Results:**

The retrospective/prospective incidence of Raynaud's phenomenon was 16/14 per 1000 exposure years among exposed and 2.4/5.0 per 1000 years among the not exposed. The retrospective dose response curve based on 4 dose classes showed that class 2, 3 and 4 had similar response and showed higher incidence than the not-exposed. The dose with RP response to hand-arm vibration corresponded to a 10 year A(8) value between 0.4–1.0 m/s^2^.

**Conclusion:**

The results indicate that the EU directive on an action value for hand-arm vibration of 2.5 m/s^2 ^is not too low. Rather, it suggests that employers should take on actions even at exposure values of 1 m/s^2^A(8).

## Background

Raynaud's phenomenon (RP) is cold provoked episodes of well-demarcated distal blanching (whiteness) in one or more fingers [1,2]. It occurs idiopathic more often among women than men [3]. Vibration induced white finger (VWF) is defined as first appearance of RP after start of professional exposure to hand-arm vibration and no other probable causes of RP [1,2]. The pathogenic mechanism of VWF is not completely understood but digital artery vasospasm is a probable cause. Both central and local mechanisms have been suggested for this vasospasm. The central mechanism may be an overactivity of the central sympathic nervous system and the local a digital vascular fault [1]. Anamnestic diagnostics by medical interview and questionnaire are widely accepted [1]. Cold induced digital artery vasospasm can also be measured by cold provocation tests [1].

Despite the number of studies published concerning VWF, the form of the exposure-response relationship for VWF is not yet clear. Most epidemiologic studies have been cross-sectional studies and have shown consistently a relation between exposure to hand-arm vibration and VWF [4,5]. The longitudinal studies have been repeated cross-sectional studies or retrospective designs [6-8]. The longitudinal studies have mostly been retrospective asking the worker whether finger blanching occurs and the date of the first occurrence [7,8]. The repeated cross-sectional studies have mostly followed the prevalence [6]. Few truely prospective studies have been reported. Bovenzi and co-workers [9] reported a 5 year prospective study of 68 forestry workers where 3 new cases of VWF were found. Interestingly the current risk assessment and the basis for work-place interventions of VWF is to a large extent based on cross-sectional studies that the annex to the ISO 5349-1 standard [10] rely on [7,11]. These studies were also one important base for the new European Union directive for hand-arm vibration [12]. The European Union directive on hand-arm vibration has set an action limit value of 2.5 m/s^2 ^(daily exposure value during 8 hours "A(8)") [12]. If the action limit is exceeded the employer has to determine and assess the risks, make provisions at avoiding or reducing exposure, provide worker information and training, encourage consultation and participation of workers and offer health surveillance [12]. The European Directive exposure standard has largely been developed from cross-sectional studies that extrapolate incidence risk from the retrospective histories of "survivors". The question arises whether the risks found in cross-sectional or retrospective cohort studies can be replicated in prospective designs. This obviously raises questions about the appropriateness of the cross-sectional approach, and so of the standard. One attempt to validate the approach would be to compare prospective analysis of exposure – response to retrospective analysis of exposure-response in the same cohort.

The objective of the present study was to assess the incidence of Raynaud's phenomenon in relation to hand-arm vibration exposure in a cohort consisting of male office and manual workers.

## Methods

Research ethical approval was obtained from Umeå University Hospital Ethical Committee.

### Cohort

The cohort consisted of male office workers and male manual workers, all full-time employed with monthly salary at an engineering plant that constructed and manufactured paper and pulp machinery. The date of enumeration of the source population was 31^st ^of December 1986 according to the plants payroll roster. There was staggered recruitment into the study, the baseline being for 148 subjects 31/12/1986 and for 93 subjects 31/01/1992. For the 1992 baseline, the payroll roster of January 31^st^, 1992 was used. Occupations among the 500 office workers were salesmen, managers, engineers, secretaries and economic clerks. From the roster 1986, 61 male office workers were randomly invited into the study, only three declined to enter the study. All the male 112 manual workers at the plant were invited to participate (there was only 2 females employed as manual workers). Occupations among the 112 manual workers were mainly welders, grinders, turners and steel platers. At the baseline examination in February 1987, 93 of the manual workers were available for invitation when an upper age limit of 55 years was set for inclusion. Three manual workers declined to enter the study. From this group 90 manual and 58 office workers were examined and entered in the cohort 1987. In 1992, additional 57 manual workers that had been employed after 1986 and additional 36 randomly invited office workers were examined and added to the cohort (none of invited declined). Follow ups were done 1992, 1997 and 2002. Thus base line consisted of 241 subjects. The five year follow up consisted of 229 subjects (loss from baseline 5%). The 10-year follow up 201 subjects (loss from baseline 17%). The 15 year follow up consisted of 114 subject (loss from baseline 23%, the baseline for 15 year follow up was the 148 subjects examined 1986). At baseline and at follow up a questionnaire was answered at the time for a medical examination. The baseline and the follow up investigations were all performed during the months February-March of the years 1987, 1992, 1997 and 2002. During this calendar period it is winter time in Sundsvall with snow and temperature outside below zero Celcius (C). The average outside temperature during February is -5 C.

### Raynaud'sphenomenon

Raynaud's phenomenon (white fingers) RP was defined as having answered yes to the question "Do you have white (pale) fingers of the type that appears when exposed to damp and cold weather"; the distribution of blanching was recorded on a hand diagram as well as the year of onset [1,2]. To accommodate workers with symptoms of coldness in hands or other type of discolouring also a question addressed coldness in hand and fingers. Answer to this question was not analyzed. All workers were also examined by a physician (TN) taking the history and performing a physical examination. Workers diagnosed with possible other diseases that could influence RP were excluded from the cohort (6 cases with carpal tunnel syndrome (CTS) confirmed with neurography and one case with cytotoxic treatment).

### Retrospective and prospective definitions in the cohort

In the retrospective cohort analysis time at risk was computed as the time from the age of 16 years until event (year of baseline or follow up questionnaire subtracted by the number of years with reported symptoms) or to being censored (last follow up without symptoms). The time to first occurrence of symptoms was regardless of side (right or left hand). The prospective cohort included all workers being without RP at baseline. Time to event was computed as the number of years from baseline to the first follow up with RP (5, 10 or 15 years) or to being censored (still no RP at last follow up). Thus a worker to be included in the prospective analysis had to be a "no case" at the base line. The "prospective" cases were included in the retrospective analysis but for these cases date of onset was their self report on RP occurrence. For the same cases in the prospective analysis the date of onset was set to the date of follow-up. Healthy workers that participated in at least one follow up were included in both the retrospective and the prospective analysis. All hand-arm vibration exposed cases had their hand-arm vibration exposure prior to the onset of RP thus the RP in hand-arm vibration exposed workers met the criteria of VWF. In the following we will term the condition RP in the workers regardless if they were exposed to hand-arm vibration or not.

### Exposure assessment

The subjective assessments of daily exposure time were collected in three ways, by diary, questionnaire and interview. In the diary, the workers were asked to note, every evening, the use of hand-held tools during the day (minutes). Furthermore, they also noted which type of tool was used and what type of work they had done. Measurement of tools was done according to ISO standard on a large number of workers [10,13]. These diaries were used for a period of two weeks and between 55% and 80% of the workers completed this investigation during each study period within 3 months after the baseline and follow ups. All the subjects also answered a questionnaire on hand-arm vibration exposure, where information was collected about the onset of hand-arm vibration exposure, exposure, duration of exposure per day and number of years of such exposure. Hand-arm vibration dose was in this study defined as the product of self-reported exposure hours and the hand-arm vibration exposure value. This is in accordance with recommendation for evaluation dose-exposure relationships for VWF [8]. Also leisure time exposure (hobbies, snowmobiling, motorcycling etc) was included in this measure based on interviews. Example, a welder using a grinder 3 hours per day and a chisel hammer 30 minutes per day for 7 years at exposure values of 6 m/s^2 ^respectively 9 m/s^2 ^got the dose of 7 years · 220 days · 3 hours · 6 m/s^2 ^= 27720 h · m/s^2 ^**+ **7 years · 220 days · 0.5 hours · 9 m/s^2 ^= 6930 h · m/s^2 ^thus the total dose of 34650 h · m/s^2^. An exposure dose of 1600 h · m/s^2 ^or less was defined as not exposed. In the retrospective analysis not exposed were office workers but in the prospective analysis also previously exposed workers that had a job transfer to office work were classified as not exposed. Among those exposed an arbitrary division into quartiles were done. Thus 5 classes of hand-arm vibration dose were obtained (unit = h · m/s^2^). Class 0 was the not exposed. In the retrospective analysis, class 1 was 1601 to 7578 h · m/s^2^, exposure class 2 was 7579–16787 h · m/s^2^, class 3 was 16788–39699 h · m/s^2^, and exposure class 4 was >39700 h · m/s^2^. In the prospective cohort dose was computed for the years 1987–1992. Class 1 was 1601–3520 h · m/s^2^, class 2 was 3521–7070 h · m/s^2^, class 3 was 7071–18086 h · m/s^2^, and exposure class 4 was >18086 h · m/s^2^.

There were workers that had been exposed before 1987 and also ended exposure before 1987 this information has been taken into account since every worker has been interviewed. In the interviews we have also considered leisure time exposure from tools and other sources of hand-arm vibration e.g. snowmobiles before and after 1987. In the part of Sweden where the plant is located job change is not frequent. When welders and other manual workers finish vocational school at age approximately 18 they get employed as manual workers (well paid job) and stay as long as possible. Our interviews revealed that occupational exposure to hand-arm vibration usually started already at age 16 when most workers were in vocational school. Thus we used the age 16 as onset of exposure time. In vocational school the two last years are to a major part practice. There was no worker that got out of hand-arm vibration exposure and then returned to exposure again. However many workers left exposed jobs most of them due to VWF.

### Statistics

Cumulative incidence was computed from the number cases (RP) divided by the number of years at risk. In the retrospective analysis years at risk were from the age of 16 until event (RP) or being censored (last follow up without RP). In the prospective study the cumulative incidence was computed from the number of new cases (RP) at the follow ups divided by number of years at risk. The years at risk were from baseline to event (RP) or being censored (last follow up without RP). The method of calculating risks over a time period with changing incidence rates is known as survival analysis. It can be applied to nonfatal risks as well as to death but the approach originated from data that related to death [14]. "Survival" was defined here as the proportion of the cohort not having RP with time. The basic model for survival data to be considered is the proportional hazards model [15]. The proportional hazard is a regression method for studying risk factors in cohort studies with a longitudinal design and hazards ratios were computed by Cox regression [16].

## Results

**Figure 1:**
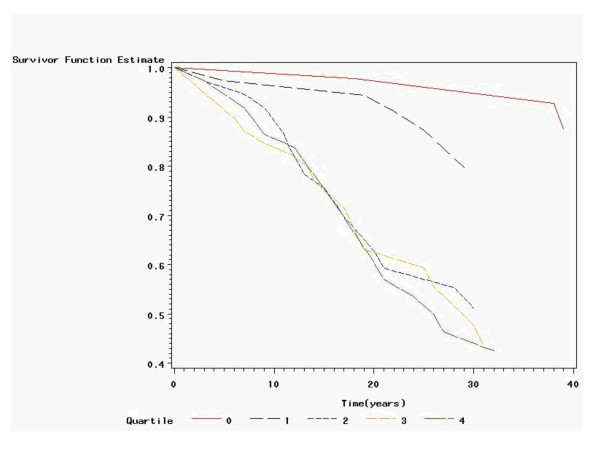
**Retrospective survival analysis of Raynaud's phenomenon in relation to exposure (hours · value (m/s^2^)**. Hand-arm vibration exposure dose was defined as the product of exposure hours and the hand-arm vibration exposure value according to ISO 5349-1 [10], unit hours · m/s^2 ^(h · m/s^2^). Non-exposed = 0–1600 h · m/s^2^, class 1 was 1601 to 7578 h · m/s^2^, exposure class 2 was 7579–16787 h · m/s^2^, class 3 was 16788–39699 h · m/s^2^, and exposure class 4 was>39699 h · m/s^2^.

The retrospective incidence of Raynaud's phenomenon was 15.9 per 1000 exposure years among exposed and 2.43 per 1000 years among the not exposed. There was a lower incidence of RP for the non-exposed compared to the hand-arm vibration exposed (Figure 1). The dose-response curve based on quartiles showed that a dose less than 7578 h · m/s^2 ^showed similar response curves as the non-exposed. The hazard ratio was for the class 2 = 10.2, class 3 = 11.4 and class 4 = 12.1 with the lower ends of confidence intervals well above one (Table 1). There were a total of 63 events and 134 being censored.

**Table 1:** Hand-arm vibration exposure in relation to retrospective Raynaud's phenomenon. Retrospective dose-response analysis Cox regression. Hand-arm vibration exposure dose was defined as the product of exposure hours and the hand-arm vibration exposure value, unit hours · m/s^2 ^(h · m/s^2^).

**Variable**	**Event**	**Censored**	**Ratio**	**95% Hazard Ratio Confidence Limits**
Non-exposed = 0–1600 h · m/s^2^	3	45	1	NA
Q1 1601–7578 h · m/s^2^	19	18	2.83	0.71–11.3
Q2 7579–16787 h · m/s^2^	19	20	10.2	2.96 – 35.0
Q3 16788–39699 h · m/s^2^	16	21	11.4	3.38 – 38.7
Q4 >39700 h · m/s^2^	6	31	12.1	3.57 – 40.9

The prospective incidence of Raynaud's phenomenon was 13.6 per 1000 exposure years among exposed and 4.97 per 1000 years among the not exposed. The prospective analysis showed no significant relation to exposure although the highest exposure class had a hazard ratio of 2.15 (Table 2). There were a total of 28 events and 157 being censored.

**Table 2:** Hand-arm vibration exposure in relation to prospective white fingers.

**Variable**	**Event**	**Censored**	**Ratio**	**95% Hazard Ratio Confidence Limits**
Non-exposed = 0–1600 h · m/s^2^	10	75	1	NA
Quartile 1 = 1601–3520 h · m/s^2^	3	22	1.02	0.28 – 3.71
Quartile 2= 3521–7070 h · m/s^2^	5	20	1.77	0.61 – 5.19
Quartile 3= 7071–18086 h · m/s^2^	4	21	1.37	0.43 – 4.382
Quartile 4 = >18086 h · m/s^2^	6	19	2.15	0.78 – 5.91

Prospective dose-response analysis Cox regression Hand-arm vibration exposure dose was defined as the product of exposure hours and the hand-arm vibration exposure value, unit hours · m/s^2 ^(h · m/s^2^).

## Discussion

The dose that displayed an increased risk of RP compared to the not exposed in the retrospective cohort analysis was in the class 7579–16787 h · m/s^2 ^corresponding to a 10 year daily exposure A(8) of about 0.4–1.0 m/s^2^. In the prospective cohort study the class above 18086 h · m/s^2 ^corresponds to a five year exposure A(8) value of about 1.0 m/s^2 ^(exposure was assessed prospectively 5 years 1987–1992). Both the results from the retrospective and the prospective cohort analysis indicate that the EU directive on an action value for hand-arm vibration of 2.5 m/s^2 ^is not too low. Furthermore, it suggests that employers may take on the proposed EU directive actions at daily exposure even at values at 1 m/s^2^.

We found another study that supports the possible hazardous effects of hand-arm vibration exposure below 1 m/s^2^. In a study of car mechanics 15% had VWF with an average daily exposure duration of 14 minutes at a value of 3.5 m/s^2 ^and an average total exposure duration of 12 years [7]. The value 3.5 m/s^2 ^for 14 minutes corresponds to an A(8) value of 0.6 m/s^2^.

Most previous research on exposure-response of hand-arm vibration and VWF was based on retrospective data where e.g. recall bias may severely affect the relationship. Our results that the cumulative incidence computed from retrospective data was similar to that of the prospective data where there were no recall biases strengthen the validity of previous research.

### Limitations in the present study

The strength in the present study is that it is both a retrospective and a prospective cohort study where the study base was taken from enrolment lists at one company with low personnel turn-over. An advantage in our study is that we only considered exposure factors until onset of symptoms. The weakness in the retrospective part of our study is that recall bias may be present among those who answered the questionnaire at the physical examination. Although we have been able to study about 10000 exposure years there were "power" problems in our study indicated by wide confidence intervals. We used the attendance date as the reference date for censoring for follow up and not the mid point between follow up. The reasons being that we wanted a valid information that RP was present furthermore the attendance date gave a more conservative value of incidence (incidence rate) compared to taking the mid point date. Another weakness in our study is the case definition. The case definition RP was based on self-report in a questionnaire, hand–diagram and physician interview. Workers with simple pallor had a possibility to report this both in the questionnaire where there was a "dummy question" about coldness in the hands/fingers and at the examination by the physician. We had no objective measurements of cold induced vasospasm. In the retrospective analysis we used historical data and assumed an onset of exposure at the age of 16. In the prospective analysis we used only confirmed data at the examinations, observed time to event and exposure values based on measurements. This may have overestimated the risks among the exposed since we did not consider exposure that had occurred before baseline measurements.

## Conclusion

- The similar cumulative incidence for the prospective and the retrospective cohort analysis supports the validity in previous studies that have used retrospective exposure and response data.

- The EU directive on an action value for hand-arm vibration of 2.5 m/s^2 ^is not too low.

- Employers are encouraged to take on the proposed EU directive actions at daily hand-arm vibration exposure even at values at 1 m/s^2^.

## Competing interests

The authors declare that they have no competing interests.

## Authors' contributions

MH wrote the manuscript, initiated and designed the study and performed the statistical analysis, interpretation and in analysis of data, LB discussed and contributed to the manuscript, designed the study, was principal investigator and data collector of the exposure measurements, participated and contributed substantial in the analysis and in the interpretation of data, RL discussed and contributed to the manuscript, designed the study, participated and contributed substantial in the analysis and in the interpretation of data, TN discussed and contributed to the manuscript, designed the study, was the examining physician at the baseline and the follow ups, participated and contributed substantial in the analysis and in the interpretation of data.
